# Nitrogen addition decreases seed germination in a temperate steppe

**DOI:** 10.1002/ece3.5151

**Published:** 2019-07-09

**Authors:** Mingxing Zhong, Yuan Miao, Shijie Han, Dong Wang

**Affiliations:** ^1^ School of Life Sciences Henan University Kaifeng China

**Keywords:** atmospheric nitrogen deposition, plant diversity, plant functional group, semi‐arid grassland, soil seed bank

## Abstract

Seed germination and seedling establishment play an important role in driving the responses of plant community structure and function to global change. Nitrogen (N) deposition is one of the driving factors of global change, which often leads to a loss in species richness in grassland ecosystems. However, how seed germination responds to N addition remains unclear. A pot incubation test was conducted in a semi‐arid grassland in the Mongolian Plateau, Northern China, to investigate the effect of N addition (0, 5, 10, 20, 40, and 80 g N/m^2^) on seed germination from May to October 2016. Twenty species germinated under all treatments; however, the responses of the 20 species to N addition were different. The densities of *Stipa krylovii*, *Leymus chinensis*, and *Artemisia frigida,* which are the dominant species in this temperate steppe, decreased significantly as the amount of N addition. Moreover, N addition significantly suppressed seedling densities of the community, perennial forbs, perennial grasses, and annuals and biennials. Furthermore, species richness of the community, perennial forbs, and annuals and biennials decreased sharply with increasing N addition level, but perennial grass species richness did not change. The Shannon–Wiener diversity index also decreased as the amount of N addition increased. Our results suggest that N enrichment plays an important role in the seed germination stage and decreases supplements of seedlings to adult plants. These findings may help explain the causes of species loss by atmospheric N deposition in grassland ecosystems.

## 1 INTRODUCTION

Anthropogenic activities, such as expansion of agriculture and fertilizer applications and combustion of fossil fuels, have significantly increased global emissions and deposition of nitrogen (N) (Battye, Aneja, & Schlesinger, 2017; Erisman et al., 2013). Numerous observational, experimental, and simulation studies have demonstrated that N enrichment has potential effects on plant communities, from alter individual plant growth to changes in plant community composition and diversity, as well as ecosystem function and services (Clark & Tilman, 2008; Reay, Dentener, Smith, Grace, & Feely, 2008; Simkin et al., 2016; Yang et al., 2012; You et al., 2017). With the current increases in N deposition, quantifying the potential responses of plant communities to increased N availability is critical for assessing future vegetation trajectories through climate–plant feedback (Isbell et al., 2013; Luo, Sherry, Zhou, & Wan, 2009).

Previous studies have focused on above‐ and below‐ground net primary production, plant diversity, and carbon cycling responses to N addition in juvenile and adult stage plants (Du et al., 2018; Humbert, Dwyer, Andrey, & Arlettaz, 2016; Pakeman et al., 2016; Soons et al., 2016; Yang et al., 2012). Many studies have shown that long‐term N addition affects offspring performance, such as seed germination, seedling establishment, and soil seed banks (Basto et al., 2015; Li, Hou, Song, Yang, & Li, 2017; Xia & Wan, 2013). The response of soil seed banks to long‐term N deposition is a major concern as they contribute to maintain in the plant community (Royo & Ristau, 2013). For example, Basto et al. (2015) report the severe negative effects of N addition on soil seed bank that threaten the stability of the plant community and recovery of the ecosystem following an anthropogenic disturbance. However, the earliest life history stage of plant growth (i.e., seed germination stage) is a central limitation in the responses of plant communities to environmental change, particularly in natural grasslands (Eriksson & Ehrlén, 1992). Changes in seed germination with N addition have the potential to affect the relative abundances of individual plants, plant community composition, and plant diversity (Varma, Iyengar, & Sankaran, 2016). Considerable gaps exist in our understanding of how seed germination responds to N deposition in grassland ecosystem.

Understanding plant species dynamics is basic to manipulating the plant community (Wu, Shang, Zhu, Ding, & Wang, 2016). The differential response of species to N addition may be a result of a change in community composition and ecosystem function through time (Isbell et al., 2013). For example, the reduction in grassland diversity in the N addition plot has been mainly determined by the loss of forbs (Foster & Gross, 1998). Nitrogen addition increases grass productivity but decreases stability of the plant community (Wang, Jmh, Brassil, & Mu, 2017; You et al., 2017). Moreover, the density of the graminoid (grasses and sedges) group increase after N addition in an alpine community (Calvo, Alonso, Fernandez, & De Luis, 2005). The various responses of plant functional groups to N addition make forecasting the response of plant communities to global change challenging. However, the time from seed germination to seedling establishment is one of the most vulnerable and crucial transitions in the life cycle of plants (He, Lv, Li, Meng, & Zhao, 2016). The responses of seed germination from different plant functional groups to N addition are controversial. For example, seed germination responses to N addition are species‐specific in a semi‐arid Mediterranean scrubland (Ochoa‐Hueso & Manrique, 2010). The seed germination proportion of N‐fixing species declines significant in responses to N addition in undrained soils, but no effect was reported in free‐draining soils (Varma et al., 2016). How the response of plant functional group to N addition are unclear at the seed germination stage.

Over the past two decades, N deposition rates have increased substantially in China (Liu et al., 2013). The semi‐arid grassland of Inner Mongolia, Northern China, has high biodiversity with important ecological functions and is an integral part of the Eurasian steppe (Kang, Han, Zhang, & Sun, 2007). Since the 1980s, this grassland has suffered over‐grazing, leading to severe loss of species and soil nutrients, making it is sensitive to N deposition (Zhang et al., 2008). The objective of this study was to determine the response of the soil seed bank to a manipulated N addition rate. In 2016, we carried out an N addition experiment in a semi‐arid grassland to examine: (a) how seedling density and diversity respond to N addition; (b) how different functional groups of seedlings respond to N addition.

## 2 MATERIALS AND METHODS

### 2.1 Site description

This study was conducted on a temperate steppe of Inner Mongolia, Northern China (42°02′N, 116°17′E, 1,324 m a. s. l.). Mean annual precipitation is approximately 382.3 mm, 90% of which falls from May to October. Mean annual temperature is approximately 2.1°C, with a range from −17.5°C in January to 18.9°C in July. The sandy soil is classified as Chestnut according to the Chinese classification (Song, Niu, & Wan, 2016). The dominant plant species in this temperate steppe are perennial grasses (*Agropyron cristatum*, *Leymus chinensis*, and *Stipa krylovii*) and forbs (*Artemisia frigida* and *Potentilla acaulis*).

### 2.2 Soil samples

Soil samples were collected from a multi‐years enclosed grassland in late April 2016, before seed germination in the field. Ten randomly selected quadrats (50 × 50 cm) were established in the grassland. Five cylindrical soil cores (8 cm diameter) were taken randomly at each of ten quadrats at a soil depth of 0–10 cm (Basto et al., 2015). All of the soil samples were mixed to obtain one combined soil sample for homogeneity. To prevent the loss of small seeds when sieving the wet soil, the soil samples were placed on a table in front of a south facing window for 10 days of direct sun exposure, and then they were sieved through mesh sieves (2 mm mesh width) to remove plant fragments and stones (Funes, Basconcelo, Diaz, & Cabido, 1999).

### 2.3 Experimental design

The samples were placed in germination trays (diameter, 20 cm, height, 16 cm) on a layer of sand that had been high temperature sterilized at 140°C for 24 hr in a dryer (Ma, Du, & Zhou, 2009). The depth of the soil layer was <3 cm. We obtained a total of 30 germination trays with soil samples. These trays were buried in the five plots with the top end of the trays 3 cm above the ground. The five plots were randomly assigned to six treatments including a control treatment and five levels of N enrichment. Nitrogen was added at 0, 5, 10, 20, 40, and 80 g N/m^2^ (0, 0.157, 0.314, 0.628, 1.256, and 2.512 g N/tray) as commercial NH_4_NO_3_. This study began on 15 May and ended on 15 October 2016. The germination trays were watered regularly with 200 ml each day.

### 2.4 Maintenance of the seed trays

The direct germination method of Thompson and Grime (1979) was used to access the composition of readily germinable seed species (Ma, Zhou, & Du, 2010). Emerging seedlings were identified and removed to maintain a low seedling density in the germination trays and to allow better germination of other seeds. At the end of the experiment, all species were divided into different functional groups based on growth form: perennial grasses, perennial forbs, annuals and biennials (Calvo et al., 2005).

### 2.5 Statistical analyses

We used the Shannon–Wiener index (*H*) and the Pielou index of evenness (*E*) to describe the seedling structural patterns.

*H* was calculated as:

(1) $$H=-\sum_{i-1}^{S}\left(P_{i}\ln P_{i}\right)$$

*E* was calculated as:

(2) $$E=\frac{H}{\ln S}$$

where *P_i_* is relative seedling abundance of species *i* and *S* is species richness of the seedlings (Wang, Zhang, Zhu, Yang, & Li, 2018).

Statistical analyses were performed using SAS version 8.0 software (SAS Institute, Cary, NC, USA). Analysis of variance with Duncan's test was used to compare the effects of different rates of N addition.

## 3 RESULTS

### 3.1 Seedling density

During the germination period, 786 (individual) seedlings from 20 species, belonging to nine families, were germinated from the soil samples. The dominant families were Poaceae and Asteraceae. Perennial grasses, perennial forbs, and annuals and biennials comprised about 58.5%, 24.6%, and 16.9% of the seedlings in this experiment. The responses of the seed bank to N addition were species‐specific (Table 1). For example, the densities of *S. krylovii*,* L. chinensis*, and *A. frigida* decreased with increasing N addition. However, *Potentilla bifurca* only geminated in the higher N addition plot.

**Table 1:** Seedling abundance of each species under different N addition treatments of the semi‐arid grassland in Inner Mongolia, Northern China

Species	Functional group	N addition (g/m^2^)					
		0	5	10	20	40	80
*Stipa krylovii*	PG	9.0 ± 0.6^a^	9.0 ± 0.4^a^	8.8 ± 0.5^a^	8.0 ± 0.5^a^	7.2 ± 0.7^ab^	6.2 ± 0.4^b^
*Leymus chinensis*	PG	7.6 ± 0.7^a^	8.0 ± 0.3^a^	6.8 ± 0.9^abc^	5.4 ± 0.8^bc^	7.0 ± 0.7^ab^	4.8 ± 0.8^c^
*Chenopodium aristatum*	AB	3.8 ± 1.9^ab^	4.6 ± 2.3^a^	2.2 ± 1.3^ab^	0.6 ± 0.4^b^	1.6 ± 1.1^ab^	0.4 ± 0.2^b^
*Melandrium apricum*	PF	2.8 ± 0.7^a^	2.2 ± 0.7^ab^	1.6 ± 0.4^ab^	0.8 ± 0.4^b^	0.6 ± 0.2^b^	0.6 ± 0.4^b^
*Artemisia frigida*	PF	2.6 ± 0.9^a^	1.2 ± 0.4^ab^	1.4 ± 0.5^ab^	1.6 ± 0.8^ab^	0.8 ± 0.4^b^	0.2 ± 0.2^b^
*Heteropappus altaicus*	PF	1.8 ± 0.7^a^	0.4 ± 0.2^b^	0.0 ± 0.0^b^	0.6 ± 0.4^b^	0.6 ± 0.4^b^	0.0 ± 0.0^b^
*Potentilla tanacetifolia*	PF	1.6 ± 0.7	2.6 ± 1.0	2.0 ± 0.7	1.4 ± 0.9	1.2 ± 0.2	0.4 ± 0.2
*Medicago ruthenica*	PF	1.2 ± 0.6	2.0 ± 0.3	1.4 ± 0.5	1.2 ± 0.6	1.8 ± 0.5	0.8 ± 0.4
*Artemisia capillaris*	PF	1.0 ± 0.8	1.0 ± 0.6	0.0 ± 0.0	0.6 ± 0.4	0.0 ± 0.0	0.0 ± 0.0b
*Artemisia dracunculus*	PF	0.8 ± 0.5	0.0 ± 0.0	0.0 ± 0.0	0.0 ± 0.0	0.8 ± 0.4	0.4 ± 0.4
*Chenopodium glaucum*	AB	0.6 ± 0.4	1.6 ± 0.9	0.8 ± 0.6	0.6 ± 0.4	1.0 ± 0.5	0.0 ± 0.0
*Artemisia sieversiana*	AB	0.2 ± 0.2	0.0 ± 0.0	0.0 ± 0.0	0.2 ± 0.2	0.2 ± 0.2	0.0 ± 0.0
*Carex korshinskyi*	PG	0.2 ± 0.2	0.0 ± 0.0	0.0 ± 0.0	0.2 ± 0.2	0.0 ± 0.0	0.0 ± 0.0
*Corispermum* *candelabrum*	AB	0.2 ± 0.2	0.0 ± 0.0	0.0 ± 0.0	0.0 ± 0.0	0.0 ± 0.0	0.0 ± 0.0
*Ixeris chinensis*	PF	0.2 ± 0.2	0.8 ± 0.4	0.0 ± 0.0	0.6 ± 0.4	0.8 ± 0.4	0.0 ± 0.0
*Cleistogenes squarrosa*	PG	0.2 ± 0.2^b^	1.4 ± 0.2^a^	0.4 ± 0.2^ab^	0.8 ± 0.5^ab^	0.4 ± 0.4^ab^	0.6 ± 0.2^ab^
*Stellaria* *media*	PF	0.0 ± 0.0	0.2 ± 0.2	0.0 ± 0.0	0.0 ± 0.0	0.0 ± 0.0	0.0 ± 0.0
*Sonchus oleraceus*	PF	0.0 ± 0.0	0.4 ± 0.2	0.4 ± 0.4	0.0 ± 0.0	0.0 ± 0.0	0.0 ± 0.0
*Allium tenuissimum*	PF	0.0 ± 0.0	0.0 ± 0.0	0.2 ± 0.2	0.4 ± 0.2	0.0 ± 0.0	0.0 ± 0.0
*Potentilla bifurca*	PF	0.0 ± 0.0	0.0 ± 0.0	0.0 ± 0.0	0.0 ± 0.0	0.2 ± 0.2	0.4 ± 0.2

Notes

Different letters following the value mean significant various among N addition gradient (Duncan's multiple range tests, *p < *0.05, *n* = 5)

AB: annuals and biennials; PF: perennial forbs; PG: perennial grasses.

**Figure 1:**
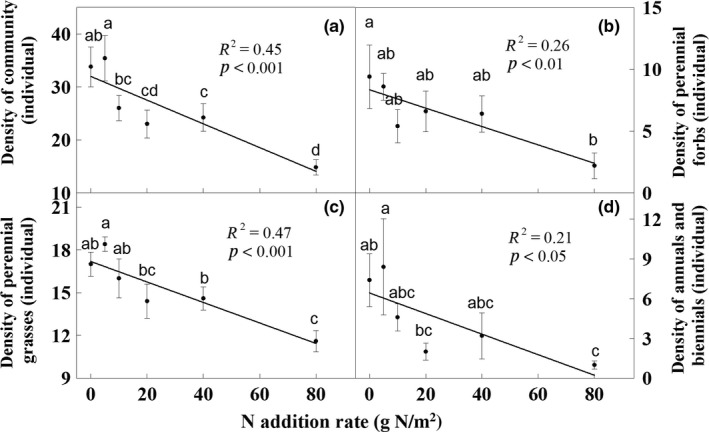
Effects of nitrogen addition on seedling density of total community (a), perennial forbs (b), perennial grasses (c), and annuals and biennials (d). Each treatment has five replicates. Different letters in the bars mean significant change among treatments by Duncan's multiple range tests (*p* < 0.05)

Nitrogen treatment had a significant effect on seedling density (*p* < 0.01, Figure 1). Seedling density decreased from 33.8 (individuals per tray, control) to 14.8 (individuals per tray, 80 g N/m^2^) across the N gradient (Figure 1). Compared to the control, mean seedling densities were suppressed by 26.6%, 31.6%, 35.0%, and 58.2%, under the 10, 20, 40, and 80 g N/m^2^ treatments, respectively (all *p* < 0.05; Figure 1). The responses of the different functional group density to the treatments were similar to those of seedling density. Nitrogen addition treatment had a significant effect on the density of perennial grasses (*p* < 0.001, Figure 1) and a slight effect on perennial forbs, and annuals and biennials (Both *p* < 0.1, Figure 1). Seedling density of all three functional groups declined linearly with an increase in the amount of N addition (Figure 1).

### 3.2 Seedling diversity

**Figure 2:**
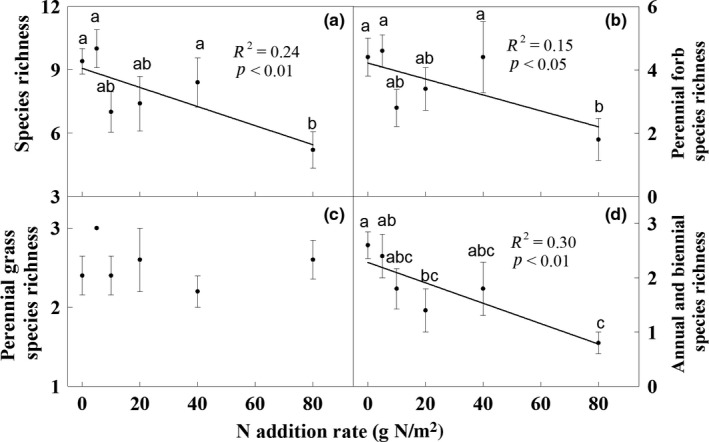
Effects of nitrogen addition rate on seedling richness of total community (a), perennial forbs (b), perennial grasses (c), and annuals and biennials (d). Different letters in the bars mean significant change among treatments by Duncan's multiple range tests (*p* < 0.05)

**Figure 3:**
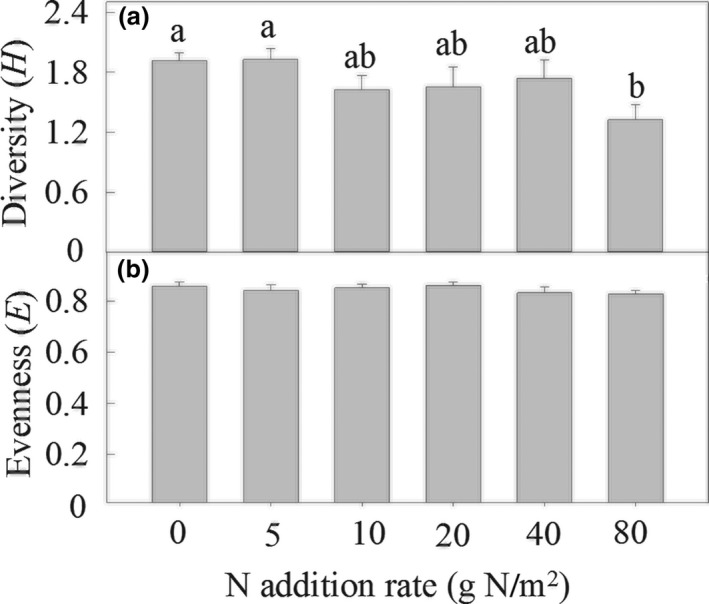
Effects of nitrogen addition rate on Shannon–Wiener index (*H*, a) and Pielou evenness index (*E*, b). Different letters in the bars means significant change among treatments by Duncan's multiple range tests (*p* < 0.05)

The N addition treatments sharply affected community species richness, and annual and biennial species richness (Both *p* < 0.05; Figure 2), but only slightly affected perennial forb species richness (*p* < 0.1; Figure 2). Species richness of the community, perennial forbs, and annuals and biennials decreased significantly by 44.7%, 59.1%, and 69.2% under the 80 g N/m^2^ treatment, respectively, compared to the ambient N treatment (Figure 2). Perennial forb species richness decreased from 5 to 2 as the amount of N addition increased (Figure 2). Nitrogen addition significantly decreased *H*, but had no effect on *E* (Figure 3).

## 4 DISCUSSION

### 4.1 Seedling density response to N addition

We found that seedling density decreased significantly as the mount of N addition increased, although the responses of seed germination to N addition were species‐specific. Our findings are inconsistent with a previous study demonstrating increased plant density with N enrichment in a grassland ecosystem (Zhou, Bowker, Tao, Wu, & Zhang, 2018). Nitrogen addition may increase asexual reproduction of plants in the adult stage (Bai, Sun, Wang, & Li, 2009). In the seed germination stage, Ochoa‐Hueso and Manrique (2010) demonstrated that germination of *Sagina apetala* Ard. was enhanced by N addition in the seed bank. Increased nitrogen availability usually acts as a signal to promote germinate of pioneer species, and complete their life cycle (Luna & Moreno, 2009), and this seemed to be the case for nitrophilous *P. bifurca *in our study. However, more previous studies have also reported that seed germination can be influenced negatively by nutrients addition (Haden et al., 2011; Kraaij & Ward, 2006; Pesch & Pieterse, 1982), which agreed with our findings. There are several explanations for the responses of seedling density to N addition. First, N addition significantly decrease soil pH in a typical steppe (Tian et al., 2016). Low soil pH depress germination (Roem, Klees, & Berendse, 2002) and plant performance (van den Berg et al., 2005). Second, soil pH not only determines nutrient availability (Chapin, 1980), but also influences the distribution of soil microbial and animal communities (Reth, Reichstein, & Falge, 2005; Shao et al., 2017), which may cause degradation of seeds by microorganisms (Chee‐Sanford, Williams, Davis, & Sims, 2006; Leishman, Masters, Clarke, & Brown, 2000) and decrease plant density. Third, nitrogen addition affects mineral ion availability in soil and physiological stress in plants, which suppresses seed germination and seedling regeneration (Roem et al., 2002; Sullivan et al., 2013), resulting in reduced of seedling density (Stevens, 2016). Additionally, N addition may increase soil metal ion concentrations, such as exchangeable manganese (Mn^2+^), ferric iron (Fe^3+^), and aluminum (Al^3+^), which are toxic to seeds and seedlings (Liu, Zhang, & Lal, 2016; Roem et al., 2002).

### 4.2 Seedling diversity response to N addition

Nitrogen addition significantly decreased species diversity of the seedlings, which was consistent with previous study demonstrated declines in species diversity with N enrichment that occur in many terrestrial ecosystems in adult stage plants (Bobbink et al., 2010; Foster & Gross, 1998; Suding et al., 2005). The differential responses of component functional groups to nutrients lead to changes in community composition (Tian et al., 2016). A species‐specific response plays an important role in the diversity response to N addition. In our study, species richness of perennial forbs, and annuals and biennials declined significantly as the amount of N addition increased, but not grasses. Different species responses to N addition may be associated with species adaptation strategies (Luna & Moreno, 2009), such as seed dormancy, life‐form, and regeneration (Finch‐Savage & Leubner‐Metzger, 2006). For example, Varma et al. (2016) observed stronger declines in the germinating proportion of the N‐fixing species under N addition treatment. Germination and survival of forbs also decrease by N addition (Foster & Gross, 1998; Ochoa‐Hueso & Manrique, 2010). The responses of seedling diversity to N addition can be explained by the following mechanism. The dominant soil processes (soil acidification, toxicity, and microbial activities) are very important factors of germination of a certain species or functional group on the seed bank (Ochoa‐Hueso & Manrique, 2010). For example, N addition significantly increases soil Mn^2+^ and Fe^3+^ concentrations, which could reduce germination of annuals (*Lactuca* *sativa*) (Liu et al., 2016; Tian et al., 2016). In the same grassland as this study, a previous paper showed that forbs have lower tolerance to Mn^2+ ^than grasses (Tian et al., 2016). Nitrogen addition also decreases soil pH, which may play a role in observed species richness differences (Stevens, Dise, Gowing, & Mountford, 2006). Nitrogen may also decrease plant diversity through nutrient imbalance (e.g., inducing P limitation), or by increasing susceptibility to diseases or pests (Phoenix et al., 2003; Power, Ashmore, & Cousins, 1998). Furthermore, N addition can reduce niche dimensions, resulting in a decrease in the number of adult stage plant species (Harpole & Tilman, 2007). The seed germination niche is also affected by environmental change (Grubb, 1977; Marques, Atman, Silveira, & de Lemos‐Filho, 2014; Vargas, Werden, & Powers, 2015). Nitrogen enrichment may increase soil nutrient concentrations, thus reducing the seed germination niche, resulting in seedling diversity loss (Grubb, 1977). However, the evenness index was no affected by N addition in our study, consistent with previous finding that species richness and evenness have different responses to nutrient enrichment (Ma, 2005). Evenness is determined by a standardized index of relative species abundance (Krebs, 1999). Moreover, previous studies have reported that light competition is a major driver of species loss following nutrient enrichment (DeMalach, Zaady, & Kadmon, 2017; Grace et al., 2016; Harpole & Tilman, 2007). The amount of light affecting the undergrowth is a principal condition controlling seed germination and seedling survival (Kolodziejek, Patykowski, & Wala, 2017). An experiment to explore the effects of the amount of light on seeds from different functional groups should be performed in the future.

We also summarized the responses of species richness to N addition according to the different grassland ecosystems from 12 studies (Table 2). Seven of the twelve studies were located in the same grassland ecosystem. All of the results showed that N addition decreases community species richness in adult stage plants. The seven studies showed that forb richness decreases in response to N addition. Nitrogen addition had a positive (one study), negative (three studies), and no effect (three studies) on grass species richness. Two studies showed that annual and biennial species richness responds negatively to N addition. These findings suggest that seedling diversity has a similar response to N addition with that of species diversity in adult stage plant. Seed germination could play an important role in the responses of community structure and composition to N addition.

**Table 2:** Summary of the community species richness and functional group (forbs, grasses, or annual and biennials) species richness responses to N addition gradient in different experiments

Framework	Site	Grassland type	N gradient (g N m^−2^ year^−1^)	N type	Total richness	Forbs	Grasses	Annuals and biennials
Tian et al. (2016)	Mongolia Plateau (42°02′N, 116°17′E)	Temperate steppe	0, 1, 2, 4, 8, 16, 32, 64	Urea	−	−	ns	
He et al. (2016)	Mongolia Plateau (42°06′N, 115°29′E)	Temperate steppe	0, 2, 5, 20, 25, 50	NaNO_3_	−			
Chen, Zhang, Mai, and Shen (2016)	Mongolia Plateau (41°44′N, 115°40′E)	Temperate steppe	0, 1.01, 4.46, 9.17, 15.76, 25.14	Urea	−	−	ns	
Xu et al. (2015)	Tibetan Plateau (33º58′N, 101º53′E)	Alpine meadow	0, 20, 40	Ammonium	−	−	+	
Zhang et al. (2014)	Mongolia Plateau (43°13′N, 116°14′E)	Temperate steppe	0, 1, 2, 3, 5, 10, 15, 20, 50	NH_4_NO_3_	−	−	−	−
Yang et al. (2012)	Mongolia Plateau (42°02′N, 116°17′E)	Temperate steppe	0, 10	Urea or NH_4_NO_3_	−	−	ns	
Song, Bao, Liu, and Zhang (2012)	Mongolia Plateau (42°02′N, 116°17′E)	Temperate steppe	0, 3, 6, 12, 24, 48	NH_4_NO_3_	−	−	−	−
Ren et al. (2010)	Tibetan Plateau (33º58′N, 101º53′E)	Alpine meadow	0, 10	Ammonium nitrate	−			
Li, Wen, Hu, and Du (2010)	Tibetan Plateau (33º58′N, 101º53′E)	Alpine meadow	0, 10.8	(NH_4_)_2_HPO_4_	−			
Bai et al. (2010)	Inner Mongolia (43°38′N, 116°42′E)	Temperate steppe	0, 1.75, 5.25, 10.5, 17.5, 28.0	NH_4_NO_3_	−			
Britton and Fisher (2007)	Eastern Highlands of Scotland (3°20′W, 57°4′N)	Heathland	0, 1, 2, 5	NH_4_NO_3_	−			
Stevens et al. (2006)	Great Britain	UK grasslands	from 0.6 to 3.6		−	−	−	

Note

“+,” positive effect; “−,” negative effect; “ns,” no significant effect.

## 5 IMPLICATIONS

Our findings show that N enrichment decreased seedling density and diversity, suggesting that seed germination is sensitive to atmospheric N deposition in grassland ecosystems. Compared to the responses of plants to N enrichment in different life stages, our results may, in part, explain the reduction of plant diversity under N enrichment. The decrease in seed germination accompanying N deposition may result in a loss of species density and species richness, further decreasing diversity in the soil seed bank. This vicious cycle will lead to the loss of additional species. Thus, further studies on the interactive effects of N deposition and light on seeds and seedlings are necessary.

## CONFLICTS OF INTEREST

None declared.

## AUTHOR CONTRIBUTIONS

Mingxing Zhong and Dong Wang conceived the idea and designed methodology; Mingxing Zhong, Yuan Miao and Dong Wang collected, analyzed the data and wrote the manuscript; and Mingxing Zhong, Shijie Han and Dong Wang contributed substantially to revisions.

## Data Availability

All data used in this paper are included in the manuscript.
